# Improving the detection of new lesions in multiple sclerosis with a cascaded 3D fully convolutional neural network approach

**DOI:** 10.3389/fnins.2022.1007619

**Published:** 2022-11-24

**Authors:** Mostafa Salem, Marwa Ahmed Ryan, Arnau Oliver, Khaled Fathy Hussain, Xavier Lladó

**Affiliations:** ^1^Research Institute of Computer Vision and Robotics, University of Girona, Girona, Spain; ^2^Department of Computer Science, Faculty of Computers and Information, Assiut University, Assiut, Egypt

**Keywords:** brain, MRI, multiple sclerosis, automatic new lesion detection, deep learning, learning-based registration, cascaded training

## Abstract

Longitudinal magnetic resonance imaging (MRI) has an important role in multiple sclerosis (MS) diagnosis and follow-up. Specifically, the presence of new lesions on brain MRI scans is considered a robust predictive biomarker for the disease progression. New lesions are a high-impact prognostic factor to predict evolution to MS or risk of disability accumulation over time. However, the detection of this disease activity is performed visually by comparing the follow-up and baseline scans. Due to the presence of small lesions, misregistration, and high inter-/intra-observer variability, this detection of new lesions is prone to errors. In this direction, one of the last Medical Image Computing and Computer Assisted Intervention (MICCAI) challenges was dealing with this automatic new lesion quantification. The *MSSEG-2: MS new lesions segmentation challenge* offers an evaluation framework for this new lesion segmentation task with a large database (100 patients, each with two-time points) compiled from the OFSEP (Observatoire français de la sclérose en plaques) cohort, the French MS registry, including 3D T2-w fluid-attenuated inversion recovery (T2-FLAIR) images from different centers and scanners. Apart from a change in centers, MRI scanners, and acquisition protocols, there are more challenges that hinder the automated detection process of new lesions such as the need for large annotated datasets, which may be not easily available, or the fact that new lesions are small areas producing a class imbalance problem that could bias trained models toward the non-lesion class. In this article, we present a novel automated method for new lesion detection of MS patient images. Our approach is based on a cascade of two 3D patch-wise fully convolutional neural networks (FCNNs). The first FCNN is trained to be more sensitive revealing possible candidate new lesion voxels, while the second FCNN is trained to reduce the number of misclassified voxels coming from the first network. 3D T2-FLAIR images from the two-time points were pre-processed and linearly co-registered. Afterward, a fully CNN, where its inputs were only the baseline and follow-up images, was trained to detect new MS lesions. Our approach obtained a mean segmentation dice similarity coefficient of 0.42 with a detection F1-score of 0.5. Compared to the challenge participants, we obtained one of the highest precision scores (PPVL = 0.52), the best PPVL rate (0.53), and a lesion detection sensitivity (SensL of 0.53).

## 1. Introduction

Multiple sclerosis (MS) is an inflammatory disease of the central nervous system and spinal cord, with its etiology remains elusive. The progression of the disease starts almost in all cases with an inflammatory syndrome in the CNS, demyelination, and axonal loss when the immune system mistakenly starts to attack the protective myelin sheath in the brain. Due to the nature of the MS disease, no drugs offer neuroprotection when progression is observed (Ther et al., 2022), although they help to decrease the myelin loss ratio. MRI imaging techniques are one of the first choices to be used in clinical practice as reported in the 2017 revision of the McDonald criteria (McDonald et al., 2001; Thompson et al., 2018), because of their ability to detect the early stages of the disease. MS is detected in patients who have not developed clinically apparent neurological disabilities 5–10 times more frequently on conventional MRI than in the clinical assessment of relapses (Sahraian and Eshaghi, 2010). MS Lesion count and volume are very important indicators for MS diagnosis and progression and have been associated with the long-term outcome of the disease (Goodin et al., 2012; Uher et al., 2017; Ouellette et al., 2018). According to Rovira et al. (2015), patients with clinical and radiological MS findings that have not been diagnosed as patients with MS must undergo a follow-up brain MRI. On longitudinal analysis, new lesions are considered a high-impact prognostic factor for MS evolution prediction and risk of disability accumulation over time (Tintore et al., 2015). Furthermore, there is a need for a lesion quantification approach for the computation of the volumetric changes in each segmented lesion between two-time points for the MS lesion evolution (Köhler et al., 2019). Manual delineation of lesion load in brain volume should be the first choice during diagnosis, but a large number of MRI slices and different scanning modalities prevent it, due to being a time-consuming procedure with large intra- and inter-rater variability (Altay et al., 2013; Egger et al., 2017). Therefore, there is an increase in the demand for automatic methods to provide fast, more robust, and reliable results, specially for the computation of lesion volumetric changes between two-time points (Köhler et al., 2019)

Many methods were proposed to automatically detect the lesion load in MRI scans (Valverde et al., 2017b; Zhang et al., 2019) and even to review the improvements in the cross-sectional field (Lladó et al., 2012; Zeng et al., 2020; Shoeibi et al., 2021). Detecting changes in longitudinal analysis for new or enlarging lesions in the follow-up scan compared to the baseline was done initially with traditional image pre-processing tools. Based on the intensity subtraction between successive time points, Sweeney et al. (2013) used logistic regression coefficients to automatically model changes over time. Also, the work of Elliott et al. (2013) incorporated both spatial and temporal information in a two-stage classifier starting with the extraction of relevant features and brain tissues and used this information to finally segment lesions. In Battaglini et al. (2014) and Ganiler et al. (2014) authors relied on thresholding the subtraction of follow-up and baseline images. By taking the changes in surrounding tissue in mind and not depending only on the intensity change, deformation field-based methods were proposed to detect lesion change (Cabezas et al., 2016; Salem et al., 2018). Relying on segmenting both time points independently, Schmidt et al. (2019) extended their work on cross-sectional (Schmidt et al., 2012) in a new pipeline to provide lesion evolution patterns. Moreover, Jain et al. (2016), based on a joint expectation-maximization (EM) framework, used the subtraction of the two-time points and cross-sectional masks of follow-up and baseline to get the longitudinal changes. Krüger et al. (2020) used a shared encoder based on a 3D CNN to process both baseline and follow-up images. The outputs of the encoders were concatenated and passed to the decoder to detect the new or enlarged lesions that appear in the follow-up images. Most traditional methods depend on the manual threshold or mask subtraction which is affected by the required registration process and could not provide results comparable to those of human raters.

The recent advance in processing methods and shift made by artificial intelligence and deep learning methods, specially convolution neural networks (CNNs) and its ability to extract features, have made them one of the first choices to implement novel approaches. For instance, the first use of CNN in MS longitudinal data was proposed by Birenbaum and Greenspan (2016) to reduce false positives after candidate selection, obtaining segmentation accuracies near to a human rater. Inspired by the work of Balakrishnan et al. (2019) to compute the deformation field (DF), Salem et al. (2020) developed a new approach to simultaneously learn the nonlinear DF between follow-up and baseline and from the learned DF and input images learn the segmentation mask. Denner et al. (2021) used the same shared encoder and different decoders to learn the tasks of segmentation and non-rigid registration. To improve the lesion map segmentation, Gessert et al. (2020) extended the 4D context by adding a temporal history and adding convGRU to aggregate the 3D representations from encoders to be passed to the decoder for the final prediction map. Despite the increased demand for new lines in longitudinal studies, work was still hindered by no reference benchmark for proposed methods. Most methods mentioned previously were trained and evaluated on in-house data or no public code was available for comparisons among methods. To overcome this limitation, the *MICCAI Multiple Sclerosis new lesion segmentation (MSSEG-2) challenge* was proposed, offering a new opportunity to progress within this research and a public performance benchmark dataset.

In this article, we present a new pipeline for automated new lesion detection of MS patient images based on a cascade of two fully convolutional neural networks (FCNNs). The first FCNN, a filter for misclassified voxels, is used to discard the vast majority of negative voxels, while the second one is used to deal with more challenging voxels that were misclassified from the first FCNN and with the high unbalancing lesion voxels compared with background, specially hard in longitudinal data due to the few change in follow-up images (i.e., few lesions). The proposed architecture builds on an initial prototype that we presented at the MSSEG-2 challenge (Commowick et al., 2021). Other works exist either in other domains as coronary calcium segmentation (Wolterink et al., 2016), liver lesions in CT scans (Christ et al., 2016), or even based on CNN models in the MS domain such as the work of Valverde et al. (2017a), which used a cascaded CNN in cross-sectional lesion detection. The proposed pipeline was trained and tested with the MSSEG-2 challenge dataset. The results were obtained using the Anima^1^ toolbox. The same measures for the challenge (detection/segmentation) are reported and compared with the rest of the participants.

## 2. Methods

The main basic block in our segmentation pipeline is the U-Net (Ronneberger et al., 2015; Çiçek et al., 2016), which proved its performance in segmentation tasks, especially in the medical area. One of the advantages the U-Net has provided to the medical community is the ability to use a small sample to create highly detailed segmentation maps, adopted in different medical applications and obtaining the best performance in medical challenges (Siddique et al., 2021). Due to its context-based learning in the two-path architecture of contracting and expansion paths, the network training is faster and provides more accurate results than other segmentation models. In this article, 3D patches were chosen to benefit from the spatial contextual information in 3D MRI and let the network deal with input of any size without the need to re-sample or resize images, which can suffer from information loss, or lesion deformation, especially in the smaller ones.

### 2.1. Cascade-based training

In general, training a model for the detection of small lesions, where the number of lesion voxels is much less than non-lesion voxels, makes the model biased to the non-lesion class. However, the problem is even more challenging in the new lesion change detection scenario, where the few changes in the follow-up images may be insufficient to train the model.

**Figure 1:**
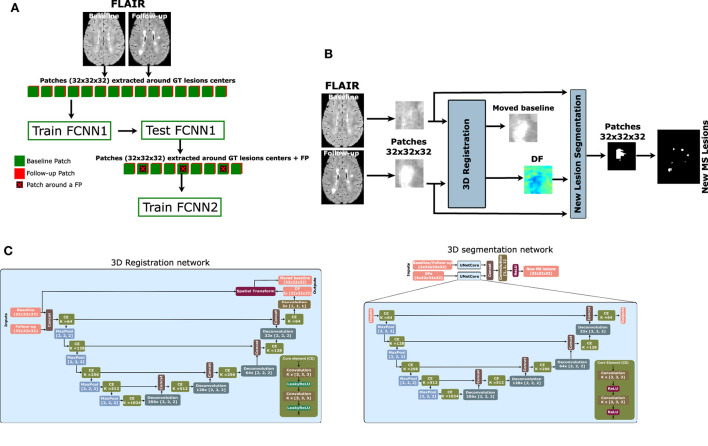
Proposed pipeline for new MS lesion detection. **(A)** Cascade-based pipeline, where the output of the first FCNN is used to select the input features of the second FCNN. **(B)** The proposed network consists of a 3D registration block and a 3D segmentation block. The inputs are baseline/follow-up images of the T2-FLAIR modality. The 3D registration block learns the deformation field (DF) and non-linearly registers the baseline image to the follow-up image. Afterward, the learned DF and the baseline and follow-up images are fed to the segmentation block, which performs the final detection and segmentation of the new lesions. The network is trained end-to-end using a combined loss function. **(C)** The 3D registration and segmentation architectures (see Salem et al., 2020 for more details).

To tackle this class imbalance problem, we propose to perform the following patch extraction strategy around the lesion voxels (see Figure 1A):

1. Extract all lesion voxels in the training images,
2. Patches of size 32×32×32 are extracted around every selected voxel in both baseline and follow-up images and stacked for the T2-w fluid-attenuated inversion recovery (T2-FLAIR) modality provided in the MSSEG-2 challenge.
3. FCNN_1_ is trained with the selected patches (details of the model available in Section 2.2).
4. Overlapped patches are extracted and tested using the trained FCNN_1_ to get the probability *Y*_1_. The probability threshold (>0.5) is used to calculate the lesion map. Also, small lesions (<3 mm^3^) are removed.
5. Based on the calculated lesion map, new patches are extracted with 32 × 32 × 32 size and step 8 × 8 × 8 around the lesion area and the misclassified lesion by FCNN_1_.
6. The second network (FCNN_2_) is trained from scratch with the newly extracted patches.
7. The output probability from the trained FCNN_1_ (*Y*_1_) is averaged with the output of the trained FCNN_2_ (*Y*_2_) to get the final lesion probability mask. To obtain the final segmentation mask, we threshold the voxel probability >0.5 and remove the small lesions (< 3*mm*^3^).

### 2.2. Network architecture

The FCNN used in our work for both FCNN_1_ and FCNN_2_ is shown in Figure 1B. It follows the most recent proposed architecture by Salem et al. (2020). The network is a fully CNN that takes the T2-FLAIR image modality in both baseline and follow-up as inputs and outputs of the new lesion segmentation mask. The network consists of two parts as shown in Figure 1C. The first part is a U-Net block that automatically learns the DF that non-linearly registers the T2-FLAIR baseline image to the follow-up space. The learned DF and the baseline and follow-up images are then fed to a second part of the network, another U-Net that performs the detection and segments of the new lesions. The network is trained end-to-end with gradient descent and simultaneously learns both DF and new lesion segmentation. This model was updated for the MSSEG-2 challenge dataset and sent to the challenge (referred to as Vicorob).

**3D registration architecture:** A 3D registration block is built for the T2-FLAIR modality following the architecture explained in Salem et al. (2020). This block is inspired by VoxelMorph, a learning framework for deformable medical image registration (Balakrishnan et al., 2019). The registration block learns the DF that non-linearly registers the T2-FLAIR baseline image to the follow-up space. It is a fully convolutional network that follows a U-shaped architecture (Ronneberger et al., 2015). The U-Net architecture consists of four downsample (the contracting path) and upsample steps (the expansive path). The core element (CE) block is a two 3D convolution layer (kernel size = 3 and stride = 1) with K channels. Each convolution is followed by a LeakyReLU layer. The number of channels, K, of CE blocks is (64, 128, 256, and 512) and (512, 256, 128, and 64) for the contracting path and expansive path, respectively. The spatial transformation (Jaderberg et al., 2015; Balakrishnan et al., 2019) warps the baseline image to the follow-up image using the learned DF and enabling end-to-end training. The LeakyReLU activations are used instead of ReLU so that the learned DFs can have both positive and negative values (see Salem et al., 2020 for more details).

**3D segmentation architecture:** A 3D segmentation CNN is also used for segmenting the new lesions. It is a two-branch network where each branch is a U-Net following the architecture explained in Salem et al. (2020). The U-Net architecture is exactly the same as the U-Net used in the registration block, but uses a ReLU activation layer instead of the LeakyReLU layer. The inputs of the first branch are the T2-FLAIR image modality in both baseline and follow-up, while the second branch input is the DF learned from the first registration block. The outputs of the two branches are concatenated before the classification step.

### 2.3. Loss functions

The loss function used in this work consists of the summation of an unsupervised and a supervised loss functions. The unsupervised loss function controls the registration part of the network (Balakrishnan et al., 2019). It consists of two components: a similarity part that penalizes differences in appearance between the moved baseline and follow-up images combined with a regularization part that enforces a spatially smooth deformation and often is modeled as a linear operator on the spatial gradients of DF, as stated in Balakrishnan et al. (2019). The supervised function, *L*_*CrossEntropy*_ (CrossEntropy), controls the segmentation part of the network and penalizes differences between the segmentation and ground truth. Therefore, the total loss function *L*_*Total*_ is:


(1) $$\begin{array}{l} L_{Total}=\underset{Segmentation\;loss\;function}{\underset{︸}{L_{CrossEntropy}(Seg,GT)}}\\ +\underset{Registration\;loss\;function}{\underset{︸}{\sum_{m\in Modalities}\left(\overset{Similarity\;part}{\overset{︷}{\frac{1}{N}\sum_{i=1}^{N}(F_{m_{i}}-B_{m}(DF_{m}{)}_{i}{)}^{2}}}+\overset{Regularization\;part}{\overset{︷}{\sum_{p\in DF}||▽DF_{m}(p)|{|}^{2}}}\right)}} \end{array}$$

where *F*_*m*_, *B*_*m*_(*DF*_*m*_), and *DF*_*m*_ are follow-up image, baseline image warped by DF (moved baseline), and DF for a modality *m*, respectively. *Seg* and *GT* are the automatic segmentation and the ground truth, respectively.

### 2.4. Model training

To adjust the weights of the cascaded pipeline, each network is trained individually. For FCNN_1_ to be more sensitive with lesion voxels candidate, patches of size 32×32×32 are extracted around lesion voxels. For FCNN_2_, the model is trained with more challenging voxels, which were wrongly classified with FCNN_1_. Patches of size 32×32×32 and step size 8×8×8 are extracted in the area of lesion voxels and incorrectly predicted lesions from FCNN_1_.

For training the pipeline, patches are extracted from the challenge's 40 patient volumes (the training set), with 25% of the selected patches used to validate the model after each epoch and to adjust the hyper-parameters. To adjust the pipeline weights, training is held for 100 epochs, with early stopping when no decrease was detected in the model validation loss after 10 epochs.

### 2.5. Model testing

**Figure 2:**
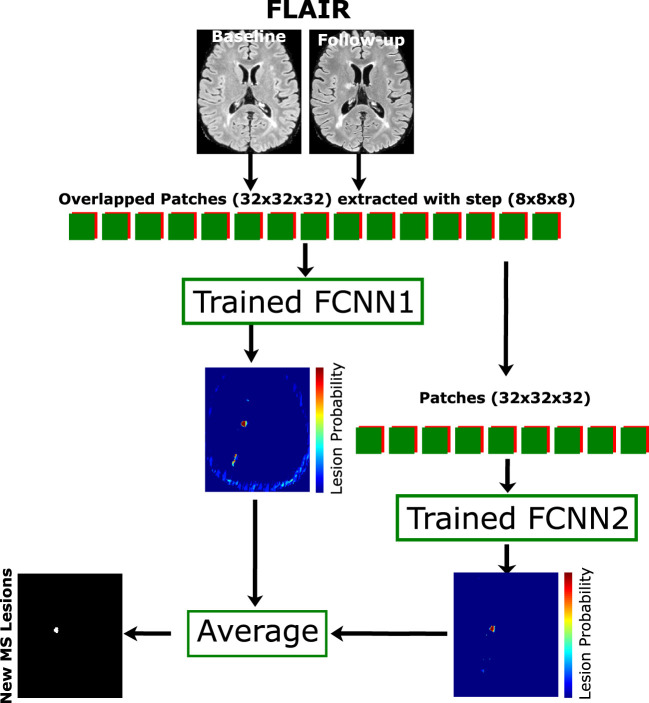
Proposed testing process. The cascade architecture of the trained network is used to segment the unseen data. Patches of size 32×32×32 are extracted from input modalities (baseline and follow-up) with step size 8×8×8 and fed to both FCNN_1_ and FCNN_2_. The average probability mask from both networks is thresholded with a minimum connected component (<3 mm^3^) to get the final lesion mask.

When the pipeline training is completed, the weights can be used with the unseen data. The overlapped extracted patches from the T2-FLAIR modality in the baseline and follow-up images and the weights of FCNN_1_ were used to get the probability P_1_, then the same extracted patches are fed to FCNN_2_ to get P_2_. The average of the two probabilities is computed and threshold by > 0.5 to get a binary mask. The final binary mask is obtained after removing the isolated voxels (region volume < 3*mm*^3^). Figure 2 shows the cascade architecture for the testing procedure.

### 2.6. Implementation details

The proposed method has been implemented in Python^2^, using Keras^3^ with the TensorFlow^4^ backend (Abadi et al., 2015). All experiments have been run on a GNU/Linux machine box running Ubuntu 18.04, with 128 GB RAM. The training was carried out on a single TITAN X GPU (NVIDIA Corp, United States) with 12 GB RAM. To promote the reproducibility and usability of our research, the proposed cascade new MS lesion detection pipeline will be available for downloading at our research website.

## 3. Experimental setup

### 3.1. Dataset

#### 3.1.1. MSSEG-2

The database used in this article is the MSSEG-2 challenge dataset. A total of 100 patients with MS were gathered. Only a 3D T2-FLAIR sequence at the first timepoint and a 3D T2-FLAIR sequence at a second timepoint (from 1 to 3 years after the first one) are available. A total of 15 different MRI scanners are represented (nine scans from three GE scanners with field strength 1.5T and 3T, 63 scans from six Philips scanners with field strength 1.5T and 3T, and 28 scans from six Siemens scanners with field strength 1.5T and 3T). The image characteristics vary with different resolutions and different voxel sizes (from 0.5 *mm*^3^ to 1.2 *mm*^3^). The gathered data are separated according to 40 scans (11 scans with no new lesions detected in the second timepoint) for training and 60 (28 scans with no new lesions detected in the second timepoint) for testing. All data from GE scanners have been excluded from the training set.

#### 3.1.2. Pre-processing

The MSSEG-2 challenge dataset is available with a rigid registration already performed to bring the two-time points of each patient to a common middle point. For each patient, the same pre-processing steps were performed on both baseline and follow-up images. First, a brain mask was identified and delineated using the ROBEX Tool (Iglesias et al., 2011). Second, the T2-FLAIR images underwent a bias field correction step using the N4 algorithm from the ITK library. Finally, the baseline and follow-up intensity values from all the training sets were normalized using a histogram-matching approach based on Nyúl et al. (2000).

### 3.2. Evaluation

The MSSEG-2 challenge performance evaluation consists of two levels as follows:

- **New lesion detection**: how many individual new lesions in the ground truth were detected by the evaluated method, independently of the precision of their contours. F1-score was chosen for this criteria.
- **New lesion segmentation**: how well are the lesions in the ground truth overlapping with those of the evaluated method. Dice measure has been selected as a score in these criteria.

The Anima^5^ toolbox, used by the challenge organizers for evaluation, is also used in all our evaluations (animaSegPerfAnalyzer). Similar to the challenge, the evaluation of lesion detection and segmentation metrics were calculated using only 32 patients from the 60 scans provided for evaluation (only patients with at least one new lesion in the follow-up). The main metric for evaluating the detection of the new lesions is the F1-score, but we also computed the precision and recall, computed as follows:


$$\begin{array}{l} F1-score=\frac{2\cdot TP}{FN+FP+2\cdot TP} \end{array}$$

$$\begin{array}{l} \mathrm{PPVL}=\frac{TP}{TP+FP} \end{array}$$

$$\begin{array}{l} \mathrm{SensL}=\frac{TP}{TP+FN} \end{array}$$

where PPVL denotes the model precision (the fraction of real lesions among the predicted ones) and SensL denotes model sensitivity or recall (the fraction of real lesions that were predicted). To evaluate the model performance in the cases with no new lesions detected at the follow-up image, the average volume (in *mm*^3^) of incorrectly predicted lesions is added to the *VolTested* measure.

The main metric to evaluate the segmentation is the dice score (DSC), which is the equivalent of the F1-score on a voxel level, and is computed as follows:


$$\begin{array}{l} DSC=\frac{2\cdot TP_{s}}{FN_{s}+FP_{s}+2\cdot TP_{s}} \end{array}$$

In segmentation, *TP*_*s*_ and *FP*_*s*_ denote the number of voxels correctly and incorrectly predicted as lesions, respectively, and *FN*_*s*_ represents the number of voxels incorrectly predicted as non-lesion.

To evaluate the significance of the obtained results, we used paired *t*-tests at a 5% level of confidence.

The following models were analyzed, aiming to show the benefits of the registration step:

- **VicorobCascade**: This is our main cascade-based model in which the registration block and segmentation block are trained simultaneously end-to-end using the loss function explained in Section 2.3. The T2-FLAIR image modality in both baseline and follow-up combined with the learned DF is fed to the segmentation block as first and second inputs, respectively.
- **DemonsDFCascade** (a.k.a. the proposed cascade-based network using the DF obtained from Demons Thirion, 1998): This model does not use the registration blocks of the proposed network shown in Figure 1B. It uses only the segmentation block with the T2-FLAIR image modality in both baseline and follow-up as the first input. The second input of the segmentation block is the DF directly computed by registering the baseline to the follow-up space for the T2-FLAIR modality using the multi-resolution Demons registration approach from ITK (Thirion, 1998). This model was used for comparison with the VicorobCascade model to highlight the impact of learned-based DF with end-to-end training over the DF from Demons.
- **NoDFCascade** (a.k.a. the proposed cascade-based network without DF): This model does not use the registration block of the proposed network shown in Figure 1B. It uses only the segmentation block with just the T2-FLAIR image modality in both baseline and follow-up as input. This model is used for comparison with the other two models to highlight the impact of the addition of the DF in increasing the detection of new lesions.

In addition to the above models, the **non-cascade** version of the three models was added to compare the normal 3D patch-based training with our proposed cascade-based training pipeline discussed in Section 2.1. Note that our original submission to the challenge is referred to here as Vicorob.

## 4. Results

Table 1 shows the F1-score, DSC, PPVL, and SensL of the proposed pipeline (VicorobCascade), the two variants (DemonsDFCascade, NoDfCascade), and the non-cascade version of each model. Results show the improvement achieved in evaluation metrics by using the cascaded-based pipeline over normal (no-cascade-based) training one. In addition, the results show the benefits of using DF and also the superiority of our cascade VicorobCascade model, where deformation fields are learned simultaneously with new lesion detection.

**Table 1:** Lesion detection and segmentation results on the MSSEG-2 challenge test set: Comparison between the different models evaluated.

**Method**	**F1-score**	**Dice**	**PPVL**	**SensL**
Vicorob	36.88 ± 29.21	35.83 ± 30.53	34.28 ± 30.22	49.80 ± 39.49
**VicorobCascade**	**49.97**±36.75	**41.97**±31.51	**51.86**±39.31	52.74 ± 39.62
DemonsDF	31.21 ± 34.68	29.08 ± 29.16	35.20 ± 39.07	36.92 ± 38.58
DemonsDFCascade	45.59 ± 35.65	41.84 ± 30.98	46.51 ± 38.55	**55.70**±37.82
NoDF	23.97 ± 30.54	25.75 ± 28.58	34.12 ± 41.95	27.84 ± 34.81
NoDfCascade	43.30 ± 34.24	39.86 ± 29.19	46.12 ± 38.55	52.43 ± 40.58

The results represent the mean F1-score, DSC, PPVL, and SensL computed by the segmentation performance analyzer tool available in Anima (animaSegPerfAnalyzer). Best values are depicted in bold.

**Figure 3:**
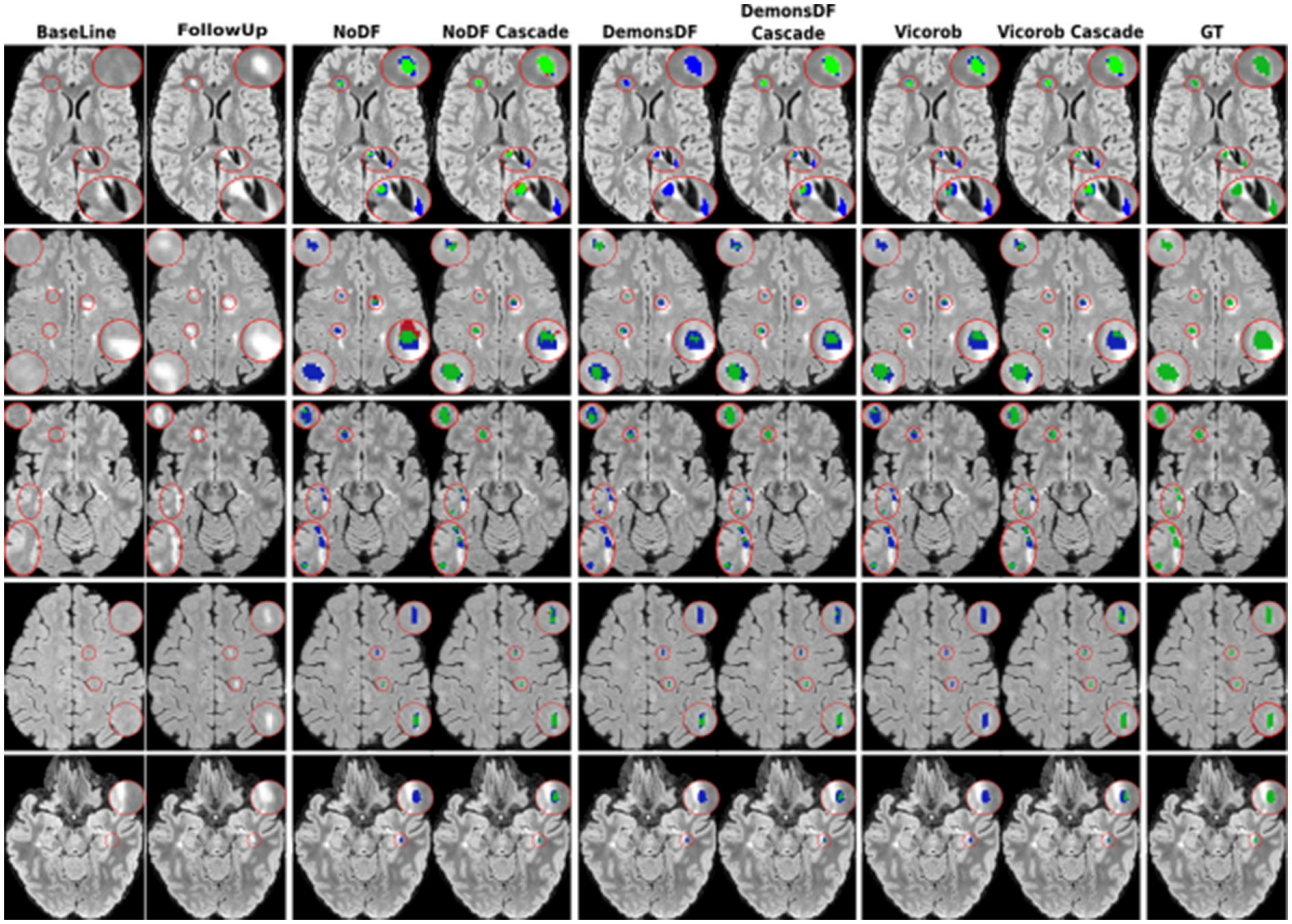
Examples of new lesion detection sensitivity improvement in axial slices. Columns correspond to baseline T2-FLAIR, follow-up T2-FLAIR and the predicted segmentation masks over follow-up T2-FLAIR for NoDF, NoDFCascade, DemonsDF, DemonsDFCascade, Vicorob, and VicorobCascade, respectively, along with the consensus ground truth (GT) mask, overlaid in green. For the predicted segmentation masks, green, red, and blue represent true positives, false positives, and false negatives, respectively.

**Figure 4:**
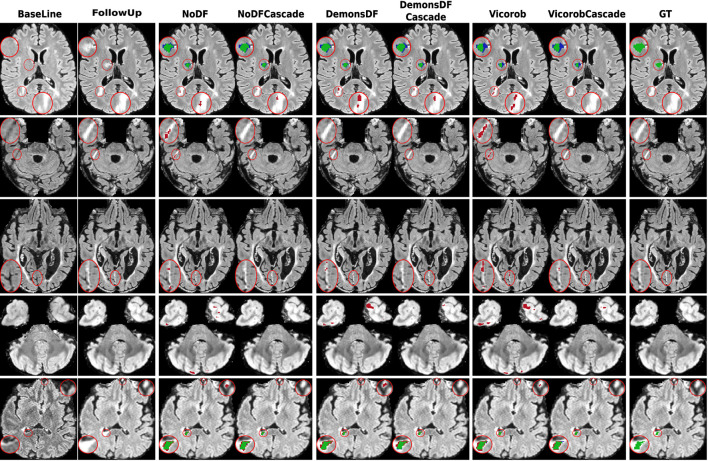
Examples of new lesion detection precision improvement in axial slices. Columns correspond to baseline T2-FLAIR, follow-up T2-FLAIR, and the predicted segmentation masks over follow-up T2-FLAIR for NoDF, NoDFCascade, DemonsDF, DemonsDFCascade, Vicorob, and VicorobCascade, respectively, along with the consensus ground truth (GT) mask, overlaid in green. For the predicted segmentation masks, green, red, and blue represent true positives, false positives, and false negatives, respectively.

Figures 3, 4 show visual examples of the improvement of the VicorobCascade model with respect to the other evaluated models. In the figures, each column corresponds to the baseline T2-FLAIR image, the follow-up T2-FLAIR image, the NoDF, NoDFCascade, DemonsDF, DemonsDFCascade, Vicorob, and VicorobCascade prediction masks, and the ground truth mask. Figure 3 shows improvement in the sensitivity of the model, while Figure 4 shows improvement in precision.

**Figure 5:**
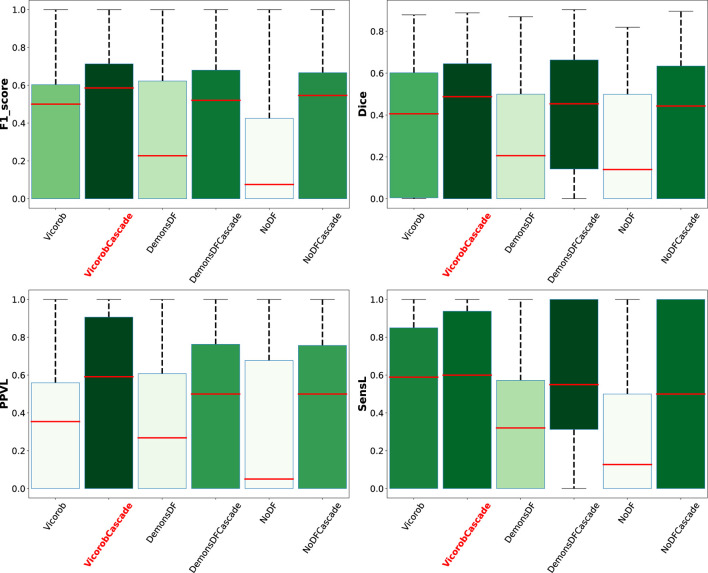
Box plot summarizing the per-patient performance of the VicorobCascade, the two variants (DemonsDFCascade, NoDFCascade), and the no-cascade-based version of the three models on the four metrics used in the evaluation (F1-score, DSC, PPVL, and SensL).

Analyzing the results per patient, Figure 5 shows a box plot summarizing the performance of the VicorobCascade, the two variants (DemonsDFCascade, NoDFCascade), and the no-cascade-based version of the three models on the four metrics used in the evaluation (F1-score, DSC, PPVL, and SensL). The results show again the superiority of the VicorobCascade over the other methods.

### Challenge results

**Figure 6:**
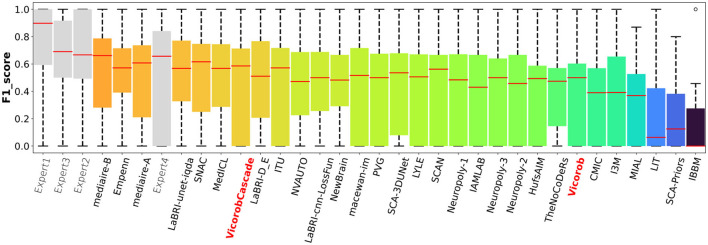
F1-score per-patient analysis. F1-score for the MSSEG-2 challenge experts, challenge teams' results, and our cascade-based pipeline (VicorobCascade).

**Figure 7:**
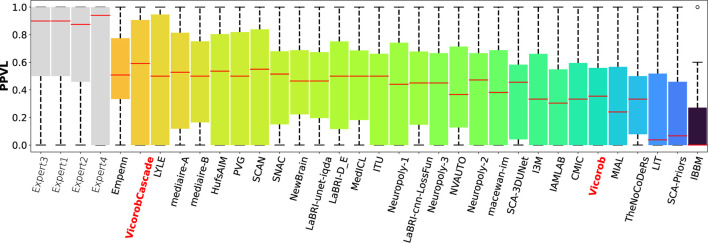
PPVL per-patient analysis. PPVL for the MSSEG-2 challenge experts, challenge teams' results, and our cascade-based pipeline (VicorobCascade). The VicorobCascade model got one of the best PPVL values between teams after the Empenn team.

The model previously submitted to the challenge under Vicorob team (referred to Vicorob) and our new cascade-based pipelines (VicorobCascade) are compared with the other challenge participants (29 pipelines for 24 teams submitted to the challenge). Figures 6, 7 show the boxplot summarizing the performance F1-score and PPVL per patient, respectively.

## 5. Discussion and future work

In this article, we have proposed a novel automated new lesions detection approach in longitudinal brain MR images. The proposed patch-wise pipeline relies on a cascade of two identical FCNNs, where the first network is trained to be more sensitive revealing possible candidate lesion voxels, while the second network is trained to reduce the number of misclassified voxels coming from the first network output. As mentioned in Salem et al. (2020), the model is trained end-to-end and simultaneously learns both the DF and the appearance of new lesions. As the DF is learned inside the network and not computed separately using classic non-rigid registration methods, the execution time of the network on a testing image is reduced compared to the time required by the state-of-the-art methods (Cabezas et al., 2016; Salem et al., 2018) from 2 to 11 min according to the test image resolution.

Regarding the end-to-end training, we trained the proposed model (VicorobCascade), two other variants (DemonsDFCascade and NoDFCascade), and the no-cascade-based version of the three models. Regarding the results without cascading, in terms of F1-score, DSC, and SensL, the Vicorob model was significantly better than all the other methods (*p* < 0.05). The F1-score improved by 5.67% compared to the DemonsDF and by 12.91% with respect to the NoDF model. In terms of PPVL, however, the performance of the Vicorob model was similar to that of the DemonsDF, although both models provided better results than the NoDF model. Notice that the model trained without any DF (NoDF) detected new lesions with a sensitivity of 27.84% and an F1-score of 23.97%. This result shows, as previously discussed in Salem et al. (2020), that the addition of DF helps to increase the detection of new lesions. However, the results also show that training the model end-to-end, simultaneously learning both the DF and the new lesions (Vicorob pipeline), performs better than using DF computed by classic deformable registration methods such as Demons (Thirion, 1998).

Regarding the cascade-based training, the proposed pipeline using two FCNN outperforms the results obtained with the baseline (no-cascade-based) approaches. The reported results show that the cascaded proposed pipeline outperformed the baseline (no-cascade-based) pipeline in all the proposed Vicorob, DemonsDF, and NoDF models for all the segmentation and detection metrics and showed also the superiority of our VicorobCascade model. The F1-score was significantly improved by 13.9%, 14.38%, and 20.85% for the Vicorob, DemonsDF, and NoDF models (*p* < 0.05), respectively. Moreover, Figure 3 shows a sensitivity improvement in the evaluated models. Notice that there is an increase in the number of true positive voxels (green ones) and decreasing in the number of false negative voxels (blue ones) between the non-cascaded and the cascaded-based models. Figure 4 shows a precision improvement for the VicorobCascade model. Notice also that there is a decrease in the number of false positive lesions compared to the other models. Regarding the cases with no new lesions, VolTested decreased from 88.40*mm*^3^ for the Vicorob model to 11.56*mm*^3^ for the VicorobCascade model.

Regarding the challenge results and compared to the challenge participants, our model (VicorobCascade) obtains one of the highest precision scores (PPVL = 0.52), the best PPVL rate (0.53), and a lesion detection sensitivity (SensL of 0.53) being superior to that of one of the challenge's human raters. Analyzing the results per scanner, the VicorobCascade model provided an F1-score of 0.22, 0.54, and 0.51 for GE, Philips, and Siemens scanners, respectively. Notice that the lower results for the GE scanner are due to the fact that data from this particular scanner were not available in the MSSEG-2 training set. Within this analysis, we also observed that the cascade-based approach obtained better results than the no-cascade one for the three scanners. Notice that there is a limitation in dealing with different image domains when data are not available. Furthermore, a clinical correlation with disability measurements could enrich the clinical evaluation of the automated segmentation results. Unfortunately, the MSSEG-2 challenge dataset does not include these clinical disability metrics. This will be taken into account in our future research work.

In conclusion, we have presented a novel approach for longitudinal analysis in patients with MS based on a cascade of two FCNNs, where the first one is able to find the potential candidates and the second one is optimized to detect new lesions and reduce the number of false positives. The obtained results indicate that the proposed end-to-end training model of the deformation fields along with the detection of new lesions combined within the cascade-based training pipeline increases the accuracy of the pipeline. Given the sensitivity and limited number of false positives, we strongly believe that the proposed method has the potential to be used in clinical studies in order to monitor the progression of the disease. We plan to release the proposed method for downloading at our research website.

## Data availability statement

The data for training and testing all the presented pipelines was obtained as part of the MICCAI 2021 MSSEG-2 challenge (https://portal.fli-iam.irisa.fr/msseg-2/data/). Access was restricted to challenge participants. Requests to access these datasets should be directed to challenges-iam@inria.fr.

## Ethics statement

The studies involving human participants were reviewed and approved by MICCAI 2021 MSSEG-2 challenge. The patients/participants provided their written informed consent to participate in this study. Written informed consent was obtained from the individual(s) for the publication of any potentially identifiable images or data included in this article.

## Author contributions

MS: thinking about the main idea, writing code, running experiments, writing the manuscript, and revision. MR: writing code, running experiments, and writing the manuscript. KH: revision. AO and XL: write the manuscript and revision. All authors contributed to the article and approved the submitted version.

## Funding

This work has been supported by DPI2020-114769RB-I00 from the Ministerio de Ciencia, Innovación y Universidades. The authors gratefully acknowledge the support of the NVIDIA Corporation with their donation of the TITAN X GPU used in this research. This work has been also supported by ICREA Academia program.

## Conflict of interest

The authors declare that the research was conducted in the absence of any commercial or financial relationships that could be construed as a potential conflict of interest.

## Publisher's note

All claims expressed in this article are solely those of the authors and do not necessarily represent those of their affiliated organizations, or those of the publisher, the editors and the reviewers. Any product that may be evaluated in this article, or claim that may be made by its manufacturer, is not guaranteed or endorsed by the publisher.

## Footnotes

^1^ https://anima.irisa.fr/

^2^ https://www.python.org

^3^ https://keras.io

^4^ https://www.tensorflow.org/

^5^ https://anima.irisa.fr/
